# A qualitative exploration of behaviours and lifestyle factors impacting levels of vitamin D within a UK ambulance service workforce (EVOLVED)

**DOI:** 10.29045/14784726.2025.9.10.2.1

**Published:** 2025-09-01

**Authors:** Emma Duncan, Theresa Foster, Larissa Prothero, Clair Hinkins, Shona Brown, Tessa Noakes, Callum Brown

**Affiliations:** East of England Ambulance Service NHS Trust ORCID iD: https://orcid.org/0000-0003-2788-8102; East of England Ambulance Service NHS Trust ORCID iD: https://orcid.org/0000-0002-6395-0885; East of England Ambulance Service NHS Trust ORCID iD: http://orcid.org/0000-0002-5440-8429; East of England Ambulance Service NHS Trust; East of England Ambulance Service NHS Trust ORCID iD: https://orcid.org/0009-0003-6510-2622; East of England Ambulance Service NHS Trust ORCID iD: https://orcid.org/0000-0001-5245-3835; East of England Ambulance Service NHS Trust ORCID iD: https://orcid.org/0009-0008-3027-026X

**Keywords:** ambulances, vitamin D, workplace

## Abstract

**Introduction:**

Vitamin D deficiency can impact health and well-being and may affect workplace performance. Shift, indoor and night working, alongside variable awareness of vitamin D, likely puts ambulance staff at an increased risk of deficiency. Screening in one ambulance service detected that 46% of staff had insufficient or deficient vitamin D levels (i.e. 50.0 nmol/L or less, as defined by NICE). The aim of the EVOLVED study was to explore the behaviours and lifestyle factors of ambulance service staff with a range of vitamin D levels and understand the impacts on their work and personal lives.

**Methods:**

A purposive sample of 40 ambulance staff was recruited over four months and invited to a one-hour online semi-structured interview. Interviews explored behaviours and lifestyle factors of those above and below the recommended adequate vitamin D levels and included questions about the impacts of vitamin D level on personal and professional well-being, with the opportunity to suggest possible improvements. Interview transcription analysis was undertaken using an intuitive thematic analysis strategy.

**Results:**

Participants were aged between 21 and 69 years and worked in varying roles, including control room (n = 9), operational (n = 20) and support staff (n = 11) and included those from diverse ethnic backgrounds to represent Trust demographics. Five themes were identified: reaction to result; diet; deficiency symptoms and impacts; impact of work on maintaining adequate vitamin D levels; and activity levels.

**Conclusion:**

A lack of awareness of vitamin D-related issues was identified, alongside a variety of improvement suggestions, including participants emphasising the importance of awareness, to allow staff to take responsibility to promote their own health and well-being. Strategies to promote awareness of vitamin D should be considered to improve staff well-being in this area. Participants positively perceived research exploring staff health and well-being, highlighting this as an area for future research.

## Introduction

Vitamin D is a fat-soluble vitamin required for calcium and phosphate regulation for the maintenance of bone, teeth and muscle health (National Health Service (NHS), 2020). Ultraviolet B rays from sunlight are the catalyst for the chemical change that enables vitamin D synthesis within the body (National Institutes of Health, 2024). Vitamin D may also be obtained from dietary sources, such as oily fish, egg yolks, red meat and liver (NHS, 2020). The National Institute for Health and Care Excellence (NICE) defines sufficient vitamin D levels as above 50 nmol/L, insufficiency as 25–50 nmol/L and deficiency as below 25 nmol/L (National Institute for Health and Care Excellence (NICE), 2022). Common signs of deficiency include fatigue, increased susceptibility to infection and illness, bone and muscle pain and depression (Amrein et al., 2020).

Indoor and shift working are risk factors for vitamin D insufficiency/deficiency (Sowah et al., 2017). Many ambulance service roles involve these factors, potentially putting ambulance staff at higher risk. Awareness of deficiency is variable among ambulance staff, further increasing their risk; the impacts of deficiency can be significant for both personal and professional well-being (Prothero & Foster, 2021). EVOLVED follows a service-screening evaluation project (VITALS), where vitamin D testing was undertaken between October and December 2022, which identified that approximately 46% of staff tested (n = 396) had below the recommended blood vitamin D levels (i.e. vitamin D less than 50.1 nmol/L). EVOLVED builds on this project by exploring the perspectives of staff participants on vitamin D-related issues.

The aim of the EVOLVED study was to explore the behaviours and lifestyle factors of ambulance staff with a range of vitamin D levels and to understand the impacts on their work and personal lives.

## Methods

EVOLVED was undertaken by researchers working for the East of England Ambulance Service NHS Trust (EEAST) with varying knowledge and experience of vitamin D. The lead researcher had previously been diagnosed with vitamin D deficiency and had experienced the impact of symptoms on both their work and personal life; this, alongside consideration of relevant guidelines and literature, guided the study design.

All EEAST staff who expressed interest in further research during VITALS (n = 396) were eligible to take part in a one-hour online semi-structured EVOLVED interview. A purposive sample of 40 participants, including those in patient-facing and office-based roles, and harder to reach groups, such as those working in Emergency Operation Centres and those from diverse ethnic backgrounds, were selected on a ‘first come first served’ basis and subsequently consented. This sample was chosen to ensure representation of Trust staff demographics and to involve participants found during VITALS to have vitamin D deficiency/insufficiency or sufficiency (as defined by NICE (2022)).

To explore lived experiences of behaviours and lifestyle factors in ambulance service staff with varying vitamin D levels, a phenomenological qualitative study design was chosen (Siriwardena & Whitley, 2022). An interpretive approach was used to include situational and personal contexts to allow for deeper exploration of staff perspectives (Lim, 2024). To enable this, and to ensure study objectives were met, semi-structured interviews were undertaken to explore staff views and experiences. These objectives included: an improved awareness of behaviours and lifestyle factors that can impact ambulance staff vitamin D levels; an improved understanding of work and personal (i.e. physical and mental health) impacts associated with vitamin D insufficiency/deficiency in ambulance staff; and suggested work-based interventions to support ambulance staff well-being in this area. The topic guide (Supplementary 1) included questions relating to the behaviours and lifestyle factors of participants, as well as questions exploring the impacts of vitamin D status on personal and professional well-being; it was reviewed by a patient and public involvement and engagement representative to ensure accessibility and applicability.

Interviews took place between 8 August and 24 November 2023 and were recorded, anonymised and audio-transcribed verbatim using Microsoft Teams. Forty interviews were proposed and took place, with this producing data saturation with little new content emerging. Transcripts were manually checked for accuracy against the recordings and analysed using an intuitive thematic analysis strategy, in line with Braun and Clarke (2022). This began with data familiarisation, with the researcher progressing to add descriptive codes to the data. Transcripts were independently coded using NVivo before iterative coding took place and a consensus was reached between researchers to reinforce intercoder reliability. Inductive reasoning was used to group codes into wider themes, with these themes then reviewed to ensure that they reflected the most prominent ideas in the data. This resulted in the adaptation of some themes, with codes compared and contrasted to ensure distinct themes were produced. These final themes were then defined and named to encapsulate all appropriate codes within the central theme. Relationships between these themes were then identified and raw data was intertwined with the researcher’s insights to produce comprehensive analysis and a variety of improvement suggestions.

## Results

The EVOLVED participant sample consisted of 26 (65%) women and 14 (35%) men, whose ages ranged from 20 to 69 years. The majority (52.5%; n = 21) had vitamin D insufficiency at the time of the VITALS study, 40% (n = 16) had vitamin D sufficiency and 7.5% (n = 3) had vitamin D deficiency. For most participants, their ethnicity was ‘White British’ (87.5%; n = 35) and their places of work included clinical operations (47.5%; n = 19), emergency operations centre (22.5%; n = 9), resilience and special operations (2.5%; n = 1) and service support (27.5%; n = 11). Further participant information is summarised in Table 1.

**Table 1.** Summary of demographic information of EVOLVED participants.

Participant number	Age range (years)	Job/role	Gender	VITALS vitamin D test result
1	50–59	Operational service delivery – patient facing	Female	Sufficient
3	30–39	Service support – office based	Female	Sufficient
5	40–49	Operational service delivery – patient facing	Male	Insufficient
7	50–59	Service support – office based	Female	Deficient
9	20–29	Operational service delivery – patient facing	Female	Sufficient
10	20–29	Operational service delivery – patient facing	Female	Sufficient
11	40–49	Operational service delivery – patient facing	Male	Insufficient
12	20–29	Operational service delivery – patient facing	Female	Sufficient
13	20–29	Operational service delivery – office based	Male	Insufficient
14	30–39	Operational service delivery – patient facing	Male	Sufficient
15	40–49	Emergency operations centre	Male	Insufficient
17	20–29	Operational service delivery – patient facing	Female	Insufficient
18	40–49	Operational service delivery – patient facing	Female	Sufficient
19	30–39	Operational service delivery – patient facing	Male	Insufficient
20	50–59	Service support – office based	Female	Sufficient
21	50–59	Operational service delivery – patient facing	Female	Deficient
22	30–39	Service support – office based	Female	Insufficient
24	30–39	Operational service delivery – patient facing	Male	Sufficient
25	50–59	Service support – office based	Female	Insufficient
28	40–49	Operational service delivery – patient facing	Male	Insufficient
29	60–69	Emergency operations centre	Female	Insufficient
30	60–69	Service support – office based	Female	Insufficient
31	30–39	Resilience and special operations	Female	Sufficient
32	40–49	Service support	Male	Insufficient
33	50–59	Service support – office based	Female	Sufficient
34	30–39	Operational service delivery – patient facing	Male	Sufficient
36	60–69	Service support – office based	Female	Insufficient
37	20–29	Operational service delivery – patient facing	Female	Insufficient
38	50–59	Service support	Male	Insufficient
40	20–29	Operational service delivery – patient facing	Female	Insufficient
41	30–39	Operational service delivery – patient facing	Female	Sufficient
42	20–29	Operational service delivery – patient facing	Female	Insufficient
43	30–39	Emergency operations centre	Male	Insufficient
44	30–39	Service support – office based	Female	Sufficient
45	30–39	Emergency operations centre	Female	Insufficient
48	40–49	Emergency operations centre	Female	Insufficient
50	40–49	Emergency operations centre	Male	Sufficient
51	30–39	Emergency operations centre	Male	Insufficient
52	40–49	Emergency operations centre	Female	Sufficient
57	50–59	Emergency operations centre	Female	Deficient

Participant numbers are not chronological, as although 57 staff members provided an expression of interest, only 40 consented and undertook an interview within the study timeframe. The reasons why participants did not progress through the study are beyond the scope of this research.

Participants reported that EVOLVED impacted them in the following ways:

- Improved knowledge:
  *I’ve learned a lot by doing it … knowing that I might be able to help myself.* (Participant 45)
- Linked staff well-being and patient outcomes:
  *It’s good to see a study being done about the staff and not the patients, because at the end of the day, if you haven’t got a healthy staff, you can’t help the patients.* (Participant 34)
- Considering how work impacts general well-being:
  *I think it’s important to recognise that the way we work does impact our lives.* (Participant 20)
- Personal positive outcomes:
  *I think it’s a really good thing … for me as an individual, it’s had a significant impact on my life.* (Participant 5)

### Theme 1: reaction to result

Sixteen participants reported being surprised or shocked by their result, thinking they were ‘*… fit and healthy and ticking along lovely [and not thinking] anything could be insufficient*’ (Participant 28).

Participants believed this stemmed from a lack of knowledge and had ‘*Not even considered [vitamin D]*’ (Participant 22). Once made aware, participants reported making changes to improve their vitamin D levels, such as ‘*trying to make some sensible or more aware lifestyle, dietary and supplementation choices*’ (Participant 19).

Most participants’ knowledge was confined to relating vitamin D and sunlight exposure. Those with poor knowledge and awareness of vitamin D had a higher incidence of insufficiency.

Those who were unsurprised by their insufficient/deficient result identified perceived risk factors for deficiency:

*Knowing the shifts that I was doing, it was expected. And so, it wasn’t a shock.* (Participant 11)

Vitamin D supplementation was not commonplace prior to VITALS, with 32 EVOLVED participants not taking supplements regularly; however, since VITALS, many participants had started taking supplements ‘*everyday rather than taking it occasionally*’ (Participant 30), albeit with mixed results:

*I did feel a lot better … I had more energy; I was more positive in myself*. (Participant 7)

*I don’t think I feel any different …* (Participant 29)

*[The supplements] actually made my bones hurt. So, I stopped taking [them].* (Participant 21)

### Theme 2: diet

Participants reported that work negatively impacted their diet, with many disclosing unhealthy eating habits:

*You just live on whatever you can get your hands on … McDonalds at 2 o’clock in the morning…* (Participant 28)

Those describing themselves as having an unhealthy diet appeared to have a higher likelihood of insufficiency.

Across a range of operational and office-based roles, a lack of breaks hindered access to healthy food at work:

*We literally go from Teams meeting to Teams meeting, without even a toilet break.* (Participant 5)

*I used to take leftover meals to heat up in the microwave, but since we’ve been queuing and so busy, we don’t get our breaks very often. So, you can’t have access to a microwave, so I don’t do that anymore.* (Participant 12)

Knowledge of good dietary sources of vitamin D was poor, with many guessing ‘*green leafy vegetables*’ (Participant 15). Of those who had poor knowledge of dietary sources, more were insufficient/deficient than sufficient:

*I knew about the sunlight exposure; I wasn’t so clued up on the dietary side of things.* (Participant 36)

Once educated on good dietary sources of vitamin D, there was varied reported consumption: ‘*Rarely. Not often*’ (Participant 33), compared to ‘*Probably quite a few times a week*’ (Participant 1). Those with a poor dietary intake had a higher likelihood of insufficiency.

### Theme 3: deficiency symptoms and impacts

Twenty-four participants reported that they had experienced symptoms of vitamin D deficiency in the past:

*… tiredness, mood just being down, you know, and really bad body aches as well ... Just felt like you’ve been hit by bus every shift.* (Participant 10)

Symptoms were often attributed to alternative causes, such as the menopause, other health conditions and shift work:

*I could tick all those things, but they’re also all the things you get when menopausal, so they’re identical.* (Participant 20)

*Fatigue, muscle aching, tiredness … you just sort of take for granted as doing shift work, maybe rather than deficient in something specific.* (Participant 40)

Symptoms were also linked to the time of year:

*I definitely become more virally unwell around autumnal winter changes.* (Participant 19)

Participants reported that deficiency symptoms affected:

*… just so many different things in life, both your physical and your mental health and although the symptoms are not sort of life changing as such, they can really impact your life.* (Participant 7)

Some participants believed that there may be organisational benefits to staff maintaining adequate vitamin D levels and suggested that ‘*address[ing] health in appraisals would be really useful*’ (Participant 37):

*The effects of it, if they can be prevented or reduced, actually staff will probably be on the road for longer because they aren’t having the aches and pains. They’re not as tired, their mental health is more reasonable...it probably helped with staff retention and things like that.* (Participant 42)

### Theme 4: impact of work on maintaining adequate vitamin D levels

Participants identified challenges in maintaining adequate vitamin D levels for ambulance staff, regardless of their role. While not all participants worked shifts, this was a repeated aspect through many responses, specifically around how shift work impacted their access to sunlight:

*You come into work in the dark, going home in the dark, you get nothing.* (Participant 45)

Regardless of working pattern, participants described ‘*not really having any significant [sunlight] exposure at all*’ (Participant 1) while at work, and when they did, this was ‘*really variable*’ (Participant 40):

*You’re either in a building or in an ambulance, and there’s very little of us that work outside, you know, regular … I would say I could go weeks without being outside whilst at work.* (Participant 21)

Some participants reported that although outside spaces were there, these were not always well maintained or appealing:

*All fenced in for security purposes ... there are cars and things coming in …* (Participant 29)

Uniform was also reported as a contributing factor to poor sunlight exposure while at work, both due to being ‘*covered from head to toe all year round*’ (Participant 21) and due to concerns around ‘*getting hot and sweaty at work*’ (Participant 17) meaning participants avoid going outside.

### Theme 5: activity levels

Those who described themselves as active were more likely to have a sufficient than insufficient/deficient result and mostly reported engaging in outdoor activities during daylight hours:

*I do try and go for a walk … and do 5–10 minute walk around the block.* (Participant 52)

Participants reported that activity levels while at work depended on their role:

*I’m quite inactive now and predominantly work from home, sitting behind a desk in front of a computer for hours on end.* (Participant 13)

Participants also reported that work impacted how active they are outside of work:

*It’s quite a physical job while you’re there anyway, so it’s 12 hours, so I don’t really want to do much more afterwards.* (Participant 40)

### Improvement suggestions

Throughout the interviews, participants made suggestions to help them achieve and maintain adequate vitamin D levels.

**Promoting healthy workplaces:**

*We need to design workplaces to be healthy … where I work actively encourages the opposite, a takeaway* *fast-food culture … sitting at your desk six hours straight, staring at five monitors.* (Participant 15)

**Improved knowledge and awareness of vitamin D:**

*Better awareness, better personal resilience, better health and well-being … it’s just education. As soon as you communicate and educate, then people can then make up their own minds.* (Participant 51)

**Improved access to healthy foods:**

*At the moment, on our welfare vehicles, we only seem to offer fizzy drinks and chocolate bars and biscuits. Maybe we could do something, especially as we are the health service, towards at least offering an alternative which would support what health is meant to be about with regards to diet and nutrition.* (Participant 5)

**Review of length and availability of breaks:**

*I think there needs to be more encouragement for people to take breaks and maybe more support for managers to allow their staff to do that. And for it to become more ingrained in the culture that we’re not here all the time that we can go out and take breaks.* (Participant 20)

*I know so many people say we need longer breaks, erm, and I think that would make a world of difference.* (Participant 29)

**Improved outside spaces:** common suggestions included ‘*communal*’ (Participant 24), ‘*green*’ (P14) areas, where staff could ‘*… actually sit down and have food*’ (Participant 1). Participants emphasised that these spaces also need to be well maintained:

*If they were created, they would start to be used, but if they’re not maintained, they’ll go to a state of disrepair.* (Participant 34)

**Ultraviolet B-emitting light bulbs:** participants believed changing lightbulbs in some areas from ‘*LED light to actually something which helps produce [vitamin D] would be a fantastic idea and it’s a simple, easy, quick fix to support our staff*’ (Participant 51).

**Informal group activities:**

*… things like organised outings, walks or things like cycling …, perhaps if we had, sort of, well-being ambassadors for each area, maybe that would be something that they could help organise.* (Participant 24)

## Discussion

Vitamin D deficiency affects up to a quarter of the UK population (Ma et al., 2021). However, a recent service-screening evaluation project (VITALS) found 46% of ambulance staff had below adequate vitamin D levels, suggesting those working for ambulance services may be at higher risk. The themes identified in EVOLVED revealed behaviours and lifestyle factors shared by ambulance service staff, which participants believed contributed to their vitamin D level; however, these should be viewed with caution due to the small sample size.

EVOLVED participants felt there should be health and well-being support from their employer, perhaps included in appraisals, to promote healthy workplaces and improve staff general health and well-being, including vitamin D levels. Participants believed their working environment put them at risk of vitamin D deficiency due to shift and indoor working reducing access to sunlight. Recent systematic reviews showed similar results, where shift and indoor workers were found to have significantly lower serum vitamin D levels compared to outdoor and non-shift workers (Martelli et al., 2022; Sowah et al., 2017). This may put ambulance staff at higher risk of complications associated with vitamin D deficiency, such as osteoporosis, fragility fractures, cancer, mental health problems and cardiovascular disease, although a lack of high-quality evidence exists for a link between vitamin D and non-musculoskeletal conditions (NICE, 2022).

In addition, shift workers are likely to have a reduced intake of vitamin D-rich foods when compared to non-shift workers (Martelli et al., 2022). EVOLVED participants had a limited knowledge of dietary sources of vitamin D but highlighted that at work they were more likely to eat unhealthy, quick and convenient foods providing little nutritional benefit. Some participants reported trying to improve their diet but work-related factors, such as availability and times of breaks, lack of facilities and a ‘fast-food culture’, were significant barriers. Only 10% of vitamin D is obtained from foods, so although diet may be an obvious area for improvement, impact to vitamin D levels may be limited, notwithstanding the impact of diet on general health and well-being (Martelli et al., 2022).

There was significant variation in the participants’ views of their results and the presumed reasons for these. Many participants were surprised and took measures to improve their vitamin D levels. Others were less surprised as they had either previously been diagnosed or had experienced deficiency symptoms. Those with poor awareness and knowledge of vitamin D had a trend towards insufficiency, aligning with Prothero and Foster (2021), where a lack of vitamin D awareness was found in ambulance staff. This is probably related to the vague nature of deficiency symptoms and that many people are asymptomatic (Holick, 2024; Tidy, 2023). Improving awareness was raised repeatedly as an important factor to empower staff to reduce their risk of deficiency and therefore reduce their risk of skeletal, infectious and chronic diseases associated with prolonged vitamin D deficiency (Holick, 2024).

Activity levels appeared to impact vitamin D levels: those who described themselves as active had a trend towards sufficiency, whereas those who described themselves as inactive trended towards insufficiency. Participants described activities mostly undertaken outdoors during daylight hours; therefore, those who undertook more activity were likely to have a higher exposure to sunlight, increasing the production of vitamin D. In addition, endurance exercise has been found to improve vitamin D levels in those who have below adequate levels; however, this varied between participant demographics (Zhang & Cao, 2022).

Those who described themselves as inactive at work trended towards insufficiency; such participants were likely to be inside during sunlight hours, reducing their sunlight exposure and vitamin D production. Participants felt that improved outside spaces at work would encourage them to spend more time outside, potentially positively impacting their vitamin D levels.

Participants suggested small but potentially influential changes, such as improved access to outside spaces and healthy foods, that could positively impact their health and well-being both at work and in their personal lives.

### Strengths and limitations

As a qualitative, exploratory study, EVOLVED was only able to identify trends and themes in the responses provided. The sample was taken from those who had previously volunteered for vitamin D testing, meaning these staff were likely to already have a higher awareness of vitamin D and may not represent the wider ambulance staff population. Forty interviews were undertaken, with results presenting staff opinions; therefore, these may not accurately represent the views of all ambulance staff. Results relied on the interpretation of the researchers, although data analysis and interviews were undertaken by the same researcher in most cases, reducing the chance for discrepancy between participant meaning and results presented.

## Conclusion

The EVOLVED study was positively received by participants and, although exploratory in nature, highlighted key behaviours and lifestyle factors of ambulance staff that may impact vitamin D levels. Participants highlighted the importance of awareness to empower staff to maintain adequate vitamin D levels. Participants valued consideration of their health and well-being by their employer and made improvement suggestions for both their work and personal lives. These will be considered by a stakeholder panel and implemented where possible. This study, alongside the preceding service evaluation, has started to raise awareness, but more could be done in this area. Future research should consider empowering staff to reduce their risk of vitamin D deficiency and to be more aware of the impact diet may have, not just on vitamin D levels, but also on general health and well-being.

## Author contributions

The study concept was devised by ED, TF, LP, TN and SB. ED, TF, LP, TN, SB and CH contributed to the topic-guide design. Interviews were conducted by ED, LP and SB, and data cleaning and analysis was overseen by ED, with assistance from LP, CH, TN, SB and CB. CH acted as a patient and public involvement and engagement representative and was involved throughout the design and delivery of the study, contributing to study management team meetings and participating in the development of study materials and data analysis. ED, with other authors providing oversight, prepared this manuscript. ED acts as the guarantor for this article.

## Conflict of interest

LP is a member of the *British Paramedic Journal* editorial board.

## Ethics

Approval from the Health Research Authority was received (reference: 23/HRA/2655). NHS Research Ethics Committee approval was not needed due to the involvement of NHS staff only. Informed consent was received from participants prior to interview completion.

## Funding

The study received funding from the Research and Development Advisory Committee (RDAC), College of Paramedics Small Grants for Research scheme.

## Use of artificial intelligence

Artificial intelligence was not used in the design, delivery or the production of results for this research.
